# Histological Features of IgA Nephropathy in Pediatrics and the Magnitude of the Disease in Saudi Children

**DOI:** 10.1155/2023/3466726

**Published:** 2023-04-03

**Authors:** Reem A. Al Zahrani

**Affiliations:** Faculty of Medicine, Department of Pathology, King Abdulaziz University and King Abdulaziz University Hospital, Jeddah, Saudi Arabia

## Abstract

**Objectives:**

This review addresses the microscopic features of immunoglobulin A nephropathy (IgA nephropathy), its prognostic variables in children, and measures to which extent these features and variables differ from adults. Furthermore, it describes the extent of this disease process among children in Saudi Arabia and the rest of the Arab countries and compares it with the data from the West and the Far East.

**Method:**

All the original work described the histological features of pediatric IgA nephropathy, and studies involved in developing the prognostic classification of IgA nephropathy, Oxford Classification, were reviewed. Moreover, the studies describing the crescent prevalence and outcome in pediatric IgA nephropathy in addition to thrombotic microangiopathy association were studied. National studies describing the prevalence of pediatric IgA nephropathy and pediatric crescentic glomerulonephritis were tracked with an overview of the regional data from the rest of the Arab world.

**Results:**

IgA nephropathy in children showed more glomerular proliferative changes and less glomerular vascular and tubule-interstitial chronic injury compared to adults. The reference study that described the association between thrombotic microangiopathy and IgA nephropathy did not include the pediatric age group. Moreover, it was found that the data from the Middle East was not encountered in developing the original and updated IgA nephropathy Oxford Classification. Furthermore, the prevalence of IgA nephropathy in children is described in the regional literature, but its histological features were not well detailed. Finally, the percentage of crescentic glomerulonephritis (GN) due to IgA nephropathy is less in our country compared to the West and concords with the Far East findings.

**Conclusion:**

A well-designed regional study addressing IgA nephropathy in Middle East children with a focus on histological features, association with crescent, and thrombotic microangiopathy and challenging the validity of the updated IgA nephropathy Oxford Classification is recommended.

## 1. Introduction

IgA nephropathy is one of the most common primary glomerular diseases worldwide and affects children and adults [1, 2]. It was initially described in 1968 by Jean Berger and Nicole Hinglais as intercapillary/mesangial glomerular deposition of IgA demonstrated by direct immunofluorescence (DIF) and was called the Berger disease [3]. Initially, this phenomenon of IgA deposition was considered a benign disease process in pediatrics [4–6]. However, further studies covered the disease course in pediatrics and proved the decrease in renal survival among patients with IgA nephropathy [7, 8]. Furthermore, a governmental screening program for urinary abnormalities started in 1974 in Japan. This program was followed by a screening-based study among pediatrics with IgA nephropathy in 1988. It confirmed that IgA nephropathy is the most common primary GN among children in the Japanese population [9]. Unfortunately, the prevalence of IgA nephropathy among the US pediatric population is not well reported [10]. In this review, we tried to extract the prevalence of IgA nephropathy and the percentage of crescentic GN due to this disease process among Saudi children by reviewing all national data describing GN and crescentic GN among pediatrics. The association of renal microvascular injury, in the form of thrombotic microangiopathy (TMA), with IgA nephropathy also requires special attention.

## 2. Method

All original work described the histological features of pediatric IgA nephropathy, and studies involved in developing the prognostic classification of IgA nephropathy, the Oxford Classification, and its validity in pediatric cases were reviewed. Moreover, the studies describing the crescent prevalence and outcome in pediatric IgA nephropathy were studied. The largest reference study described the association of TMA, and the reported cases describing this phenomenon were reviewed. Local studies describing the prevalence of GN, which included the pediatric age group, and mentioning pediatric IgA nephropathy have been tracked since the eighties. Unfortunately, none of the local studies detailed the histological features of the disease among pediatrics. Local studies that addressed pediatric crescentic GN were reviewed to estimate the proportion of crescentic GN attributed to IgA nephropathy. An overview of the regional data from the rest of the Arab world was also considered. This relatively focused literature review was performed using Google Scholar and PubMed search engines.

## 3. Histological Features in Children and the Development of Oxford Classification

What histological features of IgA nephropathy characterize the disease in children, and what correlates the most with the clinical picture? Moreover, to what extent do these histological features vary from the ones in adults? These questions were addressed in more than one study. An early multicentric study among 62 pediatric patients with IgA nephropathy was performed in 1982 in the southwest of the USA. It has been demonstrated that 40% of the patients (25/62) showed increased mesangial cellularity. 34% (21/62) showed proliferative and sclerosing GN. 26% (16/62) had essentially normal glomeruli by light microscopy. The degree of proteinuria at the time of biopsy was significantly correlated with glomerular proliferation, peripheral capillary wall changes on electron microscopy, and the presence of fibrinogen deposition by DIF. The rest of the pathological findings failed to correlate with the clinical picture statistically significantly. No significant difference between males and females was described in that study [11]. In a study performed in Japan in 2006, these differences have been well detailed by examining a comparable number of biopsies from adult and pediatric patients with IgA nephropathy. This study found that the degree of mesangial cellularity and the proportion of cellular crescent were more in the pediatric. The proportion of fibrous crescents, glomerular adhesion, and interstitial injury was more prominent in adults. Furthermore, the degree of mesangial cellularity correlated with the degree of proteinuria in the pediatrics but not in the adult [12].

In 2009, a histopathological classification of IgA nephropathy with prognostic significance was developed, the Oxford Classification. The microscopic variables of this classification included mesangial and endocapillary proliferation, segmental sclerosis, and chronic tubule-interstitial injury, i.e., interstitial fibrosis and tubular atrophy. This classification was termed as MEST score. Working groups from five continents contributed to this study. Twenty-two percent (59/265) of the patients were 18 years old or less with a similar proportion of pediatric cases ~30% from each country. This study added that the degree of endocapillary and extracapillary proliferation was more prominent in pediatrics, and vascular changes were more prominent in adults. Moreover, it supported the previously described findings of more mesangial hypercellularity and less chronic glomerular, tubular, and interstitial changes in pediatrics [13, 14]. In short, pediatric IgA nephropathy is characterized by more proliferative glomerular changes and less chronic glomerular and tubule-interstitial injury in comparison to the adult.

Although the frequency of histological variables constituting MEST score variables was quite different in pediatrics, the predictive significance of all these variables was similar among both age groups. No significant ethnic-related, histopathological, or predictive differences were described. Based on that conclusion, mesangial proliferation, endocapillary hypercellularity, segmental sclerosis, and chronic tubule-interstitial injury proved to be valid prognostic indicators in both age groups. However, it is worth mentioning that these fundamental, extensive, and well-designed studies have included a large number of participants from Europe, North America, South America, China, and Japan but did not include participants nor data from the Middle East region including Africa [13, 14].

## 4. Crescent and Crescentic Glomerulonephritis

The prognostic significance of crescent in IgA nephropathy was studied early. For instance, in a study published in 1986, 205 patients with IgA nephropathy were followed over a mean of 7.9 years. It concluded that the 10-year renal survival rate has dropped from 100% in patients with no crescent to 25.5% in patients with crescent in more than 50% of the glomeruli [15]. In 2017, an update from the IgA nephropathy classification group was issued, which recommended adding the presence and extent of the crescent as a predictive variant to the current IgA Oxford Classification and named the score as MEST-C [16]. This recommendation is based on the conclusions from different retrospective cohorts that studied 3096 patients. These studies demonstrated a decreased renal survival rate in patients with crescent in at least one glomerulus (C1 score) who did not receive immunosuppressant therapy and in patients with crescents in at least 25% of glomeruli (C2 score) [17].

In addition to that, more studies have been published addressing the predictive significance of crescent in patients with IgA nephropathy. For example, studies from the Far East have addressed the disease course in pediatric patients with IgA nephropathy and crescent. These studies emphasized the decreased renal survival rate in those patients [18, 19]. Moreover, it concluded that crescentic IgA nephropathy represented only 4.9% of the 515 studied crescentic GN cases [19].

Crescentic GN is a severe renal condition in which 50% or more of the glomeruli are involved by a crescent [20]. The causes of pediatric crescentic GN in the western region have been addressed in a study performed in Hannover in 2020. This study has found that 75% (45/60) of those cases are due to immune complex-mediated GN, and IgA nephropathy represents 42% (19/45) of this category and 32% of the total cases. The second most common cause was lupus nephritis, which caused 22% of the immune complex (10/45) and 17% of the total cases [21]. An early local study in Saudi Arabia described the causes of crescentic GN in pediatrics. It described the cases of GN with crescent among pediatrics in the country's central region over nine years from 2001 to 2009 and was published in 2011. Most cases (54.1%, 20/37) were due to lupus nephritis, which is under the immune complex-mediated GN category. IgA nephropathy fell in the rest 13% of the cases [22].

A recent local study was performed in the country's western region at King Abdulaziz University Hospital (KAUH). It studied 19 pediatric cases of crescentic GN diagnosed between 2006 and 2016. Postinfectious GN was the most common etiology (63.2%), followed by lupus nephritis, which caused 21.1% of the cases, while IgA nephropathy fell in the smallest category, representing only 5.3% of the cases [23]. Although the number of cases in this study is smaller than in the previously mentioned studies, its results concurred with the larger scale earlier study performed in the central region [22, 23]. Based on the aforementioned facts, we can conclude that IgA nephropathy is responsible for a larger proportion of crescentic GN in the West compared to our country and the Far East [19, 21–23].

## 5. Microvascular Injury (Thrombotic Microangiopathy)

The incidence of small blood vessels and capillary injury in the form of thrombotic microangiopathy in IgA nephropathy is an exciting phenomenon. Karoui et al. addressed this association in a large cohort in 2011 in France. They studied 128 patients with IgA nephropathy and found that 53% of them had associated thrombotic angiopathy. Renal survival dropped from 93.5% in patients without TMA to 52.2% in patients with TMA throughout the follow-up of 44 months. The mean patient age in this study was 38.7 years, ranging from 18–78 years. Therefore; this study may not necessarily reflect the degree of association between these two entities and the clinical behavior of such conditions in the pediatric age group, apart from being an extensive and well-designed one with valuable results [24]. Furthermore, the coincidence of atypical hemolytic uremic syndrome with IgA nephropathy was described in two different case reports, one of which was in a pediatric patient [25, 26]. Therefore, a comprehensive understanding of this phenomenon in pediatrics requires further studies describing the pathogenesis, the extent of association, clinical behavior, and the overall outcome with dedication for the pediatric age group.

## 6. Data from Local Studies

Local studies describing the GN pattern in Saudi Arabia started early in the eighties. Not all studies indicated the prevalence of IgA nephropathy in pediatrics, since some of which included adults only. It is well known that our country occupies a large area with variable population density. Most of the published studies covered the most populated region, which is the central one. Two studies covered the western area, and one only showed data from the country's eastern region, which is less populated than the central and western regions. The data related to IgA nephropathy frequency in children were extracted from the published local work chronologically that included pediatrics as listed in Table 1 [27–35].

A change in the pattern of IgA frequency in KSA has been noticed. A large cross-sectional study described the pattern of GN among pediatrics based on 326 biopsies performed in large central and western referral centers (King Khalid University Hospital and King Abdulaziz University Hospital) between January 1998 and December 2017 (Table 1, study 9). The frequency of IgA nephropathy was the lowest from 1998 to 2004 (less than 5%), followed by a 3% increase throughout 2005-2011 and then a slight drop by 1% throughout 2012-2017 [35].

Data describing the distribution and pattern of pediatric glomerular diseases in the Gulf region, including our country and other Arab, Asian, and Arab African countries, were summarized in a literature review published in 2019. It reviewed studies performed in KSA, Kuwait, Jordan, Lebanon, Sudan, Egypt, and Morocco. The frequency of IgA nephropathy was approximately more among the Asian Arab population by 5% compared to the frequency among the African Arabs. Moreover, similar to the described local studies, the frequency of IgA nephropathy is found to be the lowest among the rest of the primary glomerular diseases, such as minimal change disease and focal segmental glomerulosclerosis [36].

Local and regional studies have mainly described the crude prevalence of IgA nephropathy together with other GN in adults and pediatrics. Further, important details such as detailed microscopic findings and the extent of association with crescent and thrombotic microangiopathy were not addressed in the listed local studies [27–35]. Importantly, the prognostic validity of the IgA nephropathy Oxford Classification variables was not challenged in our region in local studies and in summary of the Arab world in both adult and pediatric age groups [27–36].

## 7. Results

IgA nephropathy in children showed more glomerular proliferative changes and less glomerular vascular and tubule-interstitial chronic injury compared to adults. The reference study that described the association between thrombotic microangiopathy and IgA nephropathy did not include the pediatric age group. Moreover, it was found that data from the Middle East was not encountered in developing the original and updated IgA nephropathy Oxford Classification. Furthermore, the prevalence of IgA nephropathy in children is described in the regional literature, but its histological features were not well detailed. Finally, the percentage of crescentic glomerulonephritis (GN) due to IgA nephropathy is less in our country compared to the West and concords with the Far East findings.

## 8. Discussion

The microscopic differences between children and adults could be summarized as more proliferative changes among pediatrics and more chronic injury changes among adults [2, 12–14]. This difference could be explained by the early disease phase in the pediatric at the time of biopsy and diagnosis before the onset of the permanent fibrosis to the kidney substructures, which may eventually occur if the disease is not well managed or detected until early adulthood. Furthermore, some of the fundamental data about the diseases that arose from the Far East region are extracted from school-based screening for renal impairment. For instance, the Japanese government established a renal function disorder screening program in 1974 [9]. This screening program allowed for the early detection of glomerulonephritis. It provided the opportunity to describe the disease's earliest phases, including the subtle, indolent, and active ones, even before it comes to the family and clinical attention. Detecting and treating the disease at this early phase allow for better control and less chronic injury. The features of chronic injury, i.e., fibrous crescent, glomerular sclerosis, interstitial fibrosis, and tubular atrophy, were more among adults [2, 12–14]. This difference could be explained by the fact that the disease process may have been going on for a while since it could be very well started in the pediatric age group and comes to the patient and clinical attention in adulthood. Also, the possible contribution of comorbidities such as obesity, hypertension, and metabolic syndromes to the overall renal outcome may play a role since these factors are more common among adults and have a considerable, well-described impact on the kidney substructures' morphology and function. The local studies described mainly the crude prevalence of IgA nephropathy among pediatrics. There is a noticeable gap in describing the detailed microscopic findings in patients with IgA nephropathy. However, these data demonstrated that it is less common than minimal change disease, focal segmental glomerulosclerosis, and kidney diseases due to systemic conditions such as lupus nephritis [27–35]. This difference in prevalence could be a true reflection of the local disease prevalence or an underestimation of higher-prevalence diseases. One thing that may support the latter theory is that there is no screening program for kidney dysfunction in our country similar to the one applied in Japan, so there may be undetected cases.

Furthermore, the local data described that renal biopsies are performed upon a clinical indication. Among the most common indications are nephrotic syndrome, subnephrotic proteinuria, unexplained kidney dysfunction, and the possibility of renal involvement in systemic diseases such as systemic lupus erythematosus; however, given the fact that clinical indication-based biopsy is the approach applied in the West is in support for that this lower prevalence is a true reflection of the disease prevalence in our country, rather than an underestimation, in addition to the concordant data from nearby regional studies [2, 12–14, 27–36]. This low disease prevalence subsequently explains the less frequency of crescentic GN due to IgA nephropathy in our region compared to the West [21–23]. This variation requires further studies to characterize the reasons behind it, which could be environmental, genetic, or geographic factors.

The exciting association of IgA nephropathy with thrombotic microangiopathy, which will at least theoretically modify the disease outcome, requires further attention among pediatrics, since it was detailed among adults only [24].

This review addressed the microscopic differences between IgA nephropathy in adults and pediatrics. Also, it sheds light on the gap in the current local and regional literature regarding pediatric IgA nephropathy.

## 9. Recommendation

Since the Middle East region, including our country, was not represented in the reference, fundamental studies, which described the variables of predictive and prognostic significance in IgA nephropathy, developed and updated the Oxford Classification of IgA nephropathy, a local and regional collaborative study challenging this classification validity in our population is recommended. Moreover, a well-designed, multicentric, and multicountry regional study is needed for IgA nephropathy in children. This study needs to address the following items: histology, the extent of association with crescent, thrombotic microangiopathy, clinical behavior, and the overall disease outcome.

## Figures and Tables

**Table 1:** List of local studies that described the prevalence of glomerulonephritis and included pediatrics or designated for pediatrics only.

	Study title	Year	Study intervals	# (%) of pediatric cases	Region	IgA nephropathy frequency
1	Qunibi et al. “Renal Disease in Saudi Arabia: A Study of 147 Renal Biopsies” [27]	1984	October 1975 until June 1983	15/147 (10%)	Central	Not mentioned
2	Akhtar et al. “Spectrum of Renal Diseases in Saudi Arabia” [28]	1990	July 1983 and August 1988	127/275 (46%)	National	7/127 (5.5%)
3	Al-Rasheed et al. **“Childhood** Renal Diseases in Saudi Arabia. A Clinicopathological Study of 167 Cases” [29]	1996	April 1988-September 1994	167	Central	3%
4	Al-Sabban et al. “Spectrum of Glomerular Disease Among Children in Saudi Arabia” [30]	1997	1981-1991	376	Central	15/376 (4%)
5	Mitwalli et al. “Glomerulonephritis in Saudi Arabia: a review” [31]	2000	January 1994 to June 1999	56/200 (28%)	Central	12/56 (21%)
6	Jalalah et al. “Childhood Primary Glomerular Diseases in the Western Region of Saudi Arabia” [32].	2009	1988 to 2006	169	Western	18/169 (10.7%)
7	Shawarbya et al. “A Clinicopathologic Study of Glomerular Disease: Experience of the King Fahd Hospital of the University, Eastern Province, Saudi Arabia” [33]	2010	1986–2008	25/233 (~11%)	Eastern	12/25 (48%)
8	Nawaz et al. “Pattern of Glomerular Disease in the Saudi Population: A Single-Centre, Five-Year Retrospective Study” [34]	2013	January 2005 to December 2009	45/176 (25.5%)	Central	11.5%
9	Alhasan et al. “Renal Histopathology Spectrum in Children with Kidney Diseases in Saudi Arabia, 1998-2017” [35]	2017	January 1998 to December 2017	326	Central and western region	17 (9.3%)

## Data Availability

All the used data are published in the literature and cited in the reference section.
